# Supplementary material for: Bioactivity Screening and Chemical Characterization of Biocompound from Endophytic Neofusicoccum parvum and Buergenerula spartinae Isolated from Mangrove Ecosystem
Source: Microorganisms. 2023 Jun 16;11(6):1599. doi: 10.3390/microorganisms11061599 (PMC10303876; doi:10.3390/microorganisms11061599)
Supplement: Supplementary file 1 [file microorganisms-11-01599-s001.zip › microorganisms-2416546-supplementary.pdf]

Fig 1.

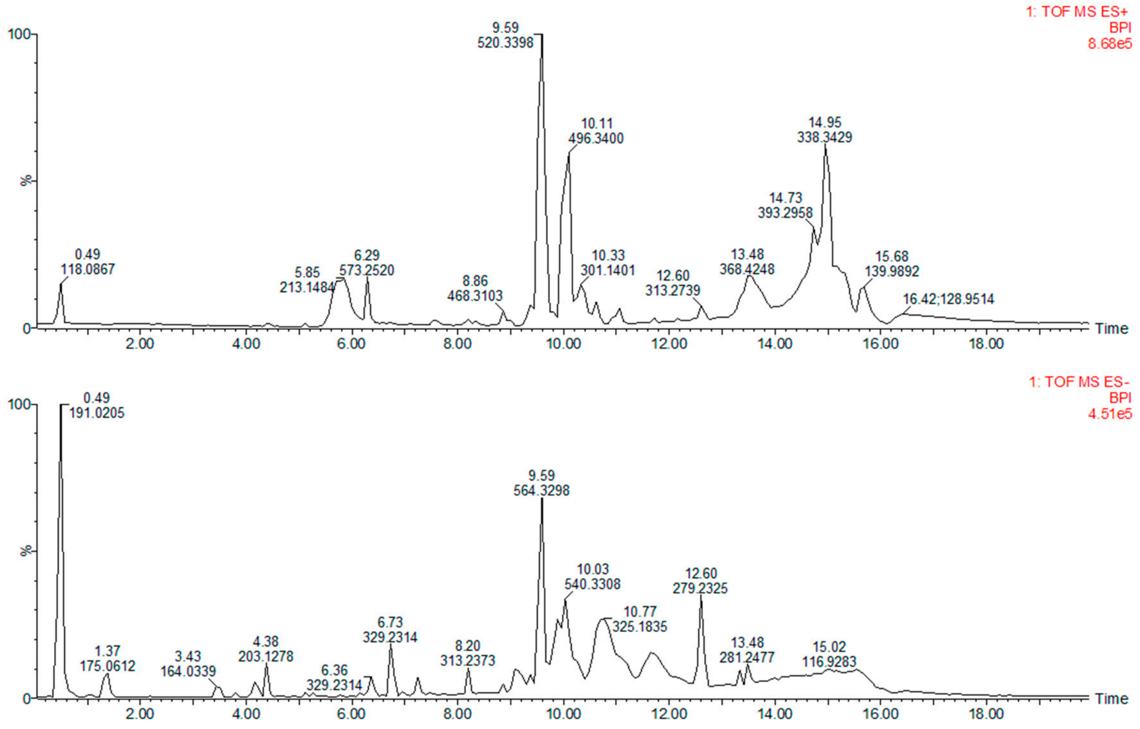

Figure S1. Ultra-Performance Liquid Chromatography-Mass Spectrometry (UPLC-MS) of crude extract from the fungus *Buergunerula spartinae* after solid-state fermentation.

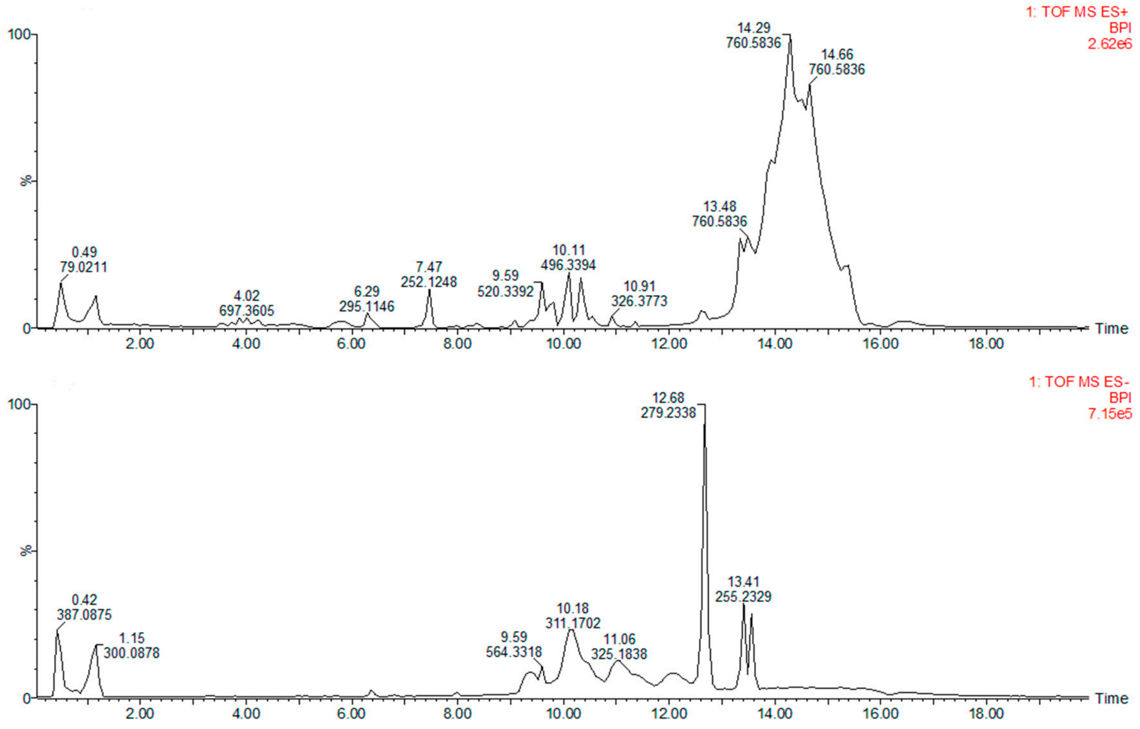

Figure S2. Ultra-Performance Liquid Chromatography-Mass Spectrometry (UPLC-MS) of crude extract from the fungus *Neofusicoccum parvum* after solid-state fermentation.
